# ST6Gal-I sialyltransferase confers cisplatin resistance in ovarian tumor cells

**DOI:** 10.1186/1757-2215-6-25

**Published:** 2013-04-11

**Authors:** Matthew J Schultz, Amanda F Swindall, John W Wright, Elizabeth S Sztul, Charles N Landen, Susan L Bellis

**Affiliations:** 1 Department of Cell, Developmental and Integrative Biology, University of Alabama at Birmingham, Birmingham, AL, USA; 2 Department of Obstetrics and Gynecology, University of Alabama at Birmingham, Birmingham, AL, USA

**Keywords:** Sialic acid, Cisplatin, Ovarian cancer, Apoptosis, Glycosylation

## Abstract

**Background:**

Platinum drugs, including cisplatin, are a frontline therapeutic in ovarian cancer treatment and acquired resistance to these agents is a major contributor to ovarian cancer morbidity and mortality. In this study a novel glycosylation-dependent mechanism for cisplatin resistance is described. Specifically, cisplatin-induced cell death is blocked by the activity of the ST6Gal-I sialyltransferase. ST6Gal-I modifies specific receptors by adding a negatively charged sialic acid sugar which influences diverse receptor functions. Overexpression of ST6Gal-I is a hallmark of ovarian and other cancers and its expression has been correlated to metastasis and poor prognosis.

**Methods:**

Tumor cell viability and apoptotic induction were determined in cell lines with ST6Gal-I overexpression and knockdown. In addition, cell populations with acquired resistance to cisplatin were assayed for endogenous ST6Gal-I expression.

**Results:**

We show that forced expression of ST6Gal-I in OV4 ovarian cancer cells that lack endogenous ST6Gal-I causes reduced activation of caspase 3 and increased cell viability following cisplatin treatment. Conversely, forced ST6Gal-I knockdown in Pa-1 cells with high endogenous ST6Gal-I increases cisplatin-induced caspase activation and cell death. A2780 ovarian cancer cells selected for stable cisplatin resistance display upregulated endogenous ST6Gal-I when compared with parental, cisplatin-sensitive, A2780 cells. Similarly, extended low dose cisplatin treatment of a Pa-1 polyclonal ST6Gal-I shRNA knockdown population led to selection for subclones with elevated ST6Gal-I expression.

**Conclusions:**

Receptor sialylation by ST6Gal-I confers a survival advantage for tumor cells in the presence of cisplatin. These collective findings support a role for ST6Gal-I in chemoresistance and highlight ST6Gal-I as a potential therapeutic target for platinum resistant tumors.

## Background

The β-galactoside α2-6-sialyltransferase ST6Gal-I catalyzes the addition of the negatively-charged sugar, sialic acid, to the termini of *N*-linked glycans on selected cell surface or secreted proteins as they transit through the Golgi. ST6Gal-I elaborates an α2-6 linkage of sialic acid to galactose, and this enzyme appears to be the primary sialyltransferase responsible for this modification in most tissues [1,2]. Depending on the specific substrate targeted by ST6Gal-I, α2-6 sialylation can modulate protein conformation, oligomerization and/or receptor internalization (reviewed in [3]). Another important function of α2-6 sialylation is to negatively regulate certain galectin-dependent cell responses [4]. Galectins are lectins that bind galactose-containing glycans, and the addition of α2-6 sialic acid to galactose impedes the ability of most galectins to bind their targets [4]. Given that many glycoprotein receptors are held on the cell surface through an interaction with the extracellular galectin lattice [5-7], ST6Gal-I-mediated sialylation can block glycoprotein binding to the lattice, causing receptor internalization. Conversely, α2-6 sialylation enhances the surface retention of other types of receptor glycoproteins [8], albeit through mechanisms not well-defined. These observations suggest that ST6Gal-I may play a role in regulating the complement of receptors on the cell surface, in addition to modulating the function of distinct glycoproteins through effects on receptor conformation and/or clustering.

ST6Gal-I is overexpressed in many different types of cancers including ovarian, breast, and colon carcinoma (reviewed in [3,4]), and ST6Gal-I upregulation is driven by oncogenic ras [9,10]. Elevated expression of ST6Gal-I has been correlated with a negative patient prognosis in breast and colorectal cancers [11,12]. Cell culture studies suggest that ST6Gal-I promotes cell migration and invasion, at least in part through altering the sialylation and function of the β1 integrin [13-15]. More recently ST6Gal-I has also been identified as an inhibitor of several cell death pathways. For example, one important function of extracellular galectins is to induce apoptosis, and this activity is blocked by ST6Gal-I mediated sialylation of galectin substrates [16-18]. Additionally, our group has shown that sialylation of the Fas and TNFR1 death receptors by ST6Gal-I hinders apoptotic signaling in response to their respective ligands, FasL and TNFα [8,19]. Finally, ST6Gal-I activity is associated with resistance to radiation treatment [20].

In view of ST6Gal-I’s upregulation in cancer, as well as its emerging role as an inhibitor of cell death pathways, we investigated whether ST6Gal-I activity could influence the sensitivity of tumor cells to cisplatin. Cisplatin is the parent compound of the platinum family of chemotherapeutics commonly used in frontline ovarian cancer treatment. Cisplatin and other platinum derivatives (e.g., oxaliplatin, carboplatin) function by forming inter- and intra-strand crosslinks in DNA, leading to an apoptotic cell death. Resistance to platinum drugs represents a major treatment challenge in ovarian and other cancers. The vast majority of ovarian cancer patients have an initial response to platinum compounds, however up to 75% of patients will relapse, with most exhibiting drug resistant disease [21]. The molecular events underlying resistance are complex, and it is likely that different tumor cells exhibit different mechanisms, or combinations of mechanisms, to escape cisplatin-induced apoptosis. At present, investigations into the mechanisms of tumor cell resistance to platinum agents have focused on drug import or export [22], cytosolic inactivation (e.g. by glutathione and other antioxidants) [23], compensatory DNA repair [24], and defects in apoptotic signaling [25]. The activation of caspases following DNA damage is important for cisplatin-induced cell death, therefore factors impinging on caspase activity can influence drug efficacy. As well, cisplatin may elicit cytotoxicity through mechanisms independent of DNA damage, as cisplatin is known to bind many molecules other than DNA, and can also modulate cytoskeletal structure [26]. In the current study we describe a new mechanism for cisplatin resistance involving α2-6 sialylation of glycoproteins by the ST6Gal-I sialyltransferase.

## Methods

### Cell lines

The Pa-1 ovarian cancer cell line was purchased commercially through ATCC (Manassas, VA). Pa-1 cells were cultured and grown in Dulbecco’s eagle's minimal essential medium (DMEM) with 4.5 g glucose supplemented with 10% fetal bovine serum (FBS)(Hyclone) and 1% antibacterial/antimycotic solution containing penicillin, streptomycin, and amphotericin B (Invitrogen). Pa-1 cells were previously found to express high endogenous levels of ST6Gal-I [13]. To examine the effects of ST6Gal-I expression on cell response to cisplatin treatment a shRNA construct targeting ST6Gal-I as well as an empty vector control were introduced via a lentiviral vector (empty vector and shRNA-expressing lentiviral particles were purchased from Sigma). Pa-1 empty vector (EV) and ST6Gal-I shRNA-mediated knockdown (sh.ST6) lines are stable, polyclonal cell populations initially selected by puromycin at a concentration of 10 μg/ml, and then maintained in 0.5 μg/ml puromycin. The OV4 ovarian cancer cell line was a generous gift from Dr. Timothy Eberlein (Harvard, Cambridge, MA). OV4 cells were cultured and grown in Dulbecco's modified Eagle's MEM/Ham's F-12 50:50 (DMEM/F12) supplemented with 10% FBS and 1% antibiotic/antimycotic solution. OV4 cells lack detectable endogenous ST6Gal-I expression and we previously forced ST6Gal-I expression and an empty vector control by lentiviral transduction (MOI = 3) [13]. Stable, polyclonal populations were isolated through puromycin selection. A2780ip2 and A2780cp20 cell lines were generous gifts from Dr. Anil Sood (MD Anderson Cancer Center). Lines were maintained in RPMI media (Cellgro) supplemented with 10% FBS and 1% antibiotic/antimycotic solution. A2780cp20 cells represent a cisplatin-resistant derivative cell line of A2780ip2 created by repeated cisplatin exposure as previously described [27].

### Immunofluorescence imaging

Cells were seeded onto 4-well chamber microscope slides (Beckin Dickinson) and allowed to adhere overnight. Cells were washed and fixed in 4% paraformaldehyde for 10 minutes followed by permeabilization in 5% Triton X-100 (in PBS) for 5 minutes. Cells were then incubated overnight at 4°C or 3 hr at room temperature with the ST6Gal-I antibody (polyclonal, R&D Systems, catalog # AF5924) and 3 hr at room temperature with anti-Golgi Matrix-130 (GM-130) (monoclonal, BD Transduction Laboratories). Following incubation with primary antibody, cells were washed and incubated with anti-goat Alexa-Fluor 594-conjugated or anti-mouse Alexa-Fluor 488 secondary antibody (Molecular Probes) for 30 minutes. Chambers were removed and DAPI-containing mounting solution, Vectamount (Vector Labs), was placed onto each well. Coverslips were added and the slides visualized under a Nikon Eclipe 80i fluorescence microscope fitted with a Photometrics CoolSNAP camera (Roper Scientific). Images were analyzed on NIS elements software. ST6Gal-I co-localization with GM-130 was imaged by confocal microscopy.

### Western blot

Prior to lysis cells were grown in puromycin free media for at least one day, passaged normally, and plated onto 6 well tissue culture plates (Fisher) at a density of 7.5 × 10^5^ cells per well. Cells were allowed to adhere overnight and then treated with cisplatin. Cells were lysed on ice in 50 mM Tris-HCl (pH 7.4) containing 1% Triton X-100, and a protease inhibitor cocktail (Roche Applied Bioscience). Cell lysate was kept on ice for 40 minutes vortexing regularly or lysates were sonicated using a sonicator model C-18 (Fisher). Protein concentrations of the lysates were determined using a modified Bradford Assay (Sigma). Proteins were then resolved by SDS-PAGE, and transferred to polyvinylidene difluoride membranes. Membranes were blocked with 5% nonfat dry milk in TBS containing 0.1% Tween 20 (TBST). Primary antibodies against ST6Gal-I (R&D Systems) or cleaved caspase-3 (Cell Signaling) were added to the membrane. Membranes were then washed and incubated with horseradish peroxidase-coupled secondary antibody (Amersham) and visualized with Immobilon enhanced chemiluminscence reagent (Millipore). Protein loading was evaluated by immunoblotting for either β-tubulin or β-actin (Cell Signaling).

### Cell viability assay

Cells were plated in opaque-sided 96-well plates (Corning) at a density of 10^4^ cells per well in 75 μl of media and allowed to adhere overnight. Cisplatin stock solutions were made by dissolving solid cisplatin in distilled water to a concentration of 2.5 mM and stored at 4°C with new solutions made monthly. On the day of each experiment, stock solutions were used to dilute cisplatin into media to obtain the desired concentrations, and then cells were grown in the cisplatin-containing media for 21 hours (Pa-1) or 24 hours (OV4). Cell viability was evaluated by determining ATP content using the CellGlo ATP quantification kit (Promega) following the manufacturer’s protocol. Luminescence was measured on a Synergy 2 plate reader (Biotek).

### Cell selection with cisplatin

The polyclonal Pa-1 sh.ST6 population contains stable clones with varying levels of ST6Gal-I knockdown. Two cell flasks were grown in parallel; one with DMEM containing 10% FBS and 1% antibiotic/antimycotic solution (control), and the other with this media supplemented with 1 μM cisplatin. After a 3-week interval, greater than 90% of the cells grown in cisplatin-containing media had died, whereas the control population proliferated over this interval. At the end of the 3 week incubation in cisplatin, the remaining viable cells were resuspended in 10% FBS/DMEM lacking cisplatin, and cultures were expanded to gain a sufficient number of cells for lysis and western blot analysis.

## Results

### Forced overexpression of ST6Gal-I confers tumor cell resistance to cisplatin-induced apoptosis

**Figure 1:**
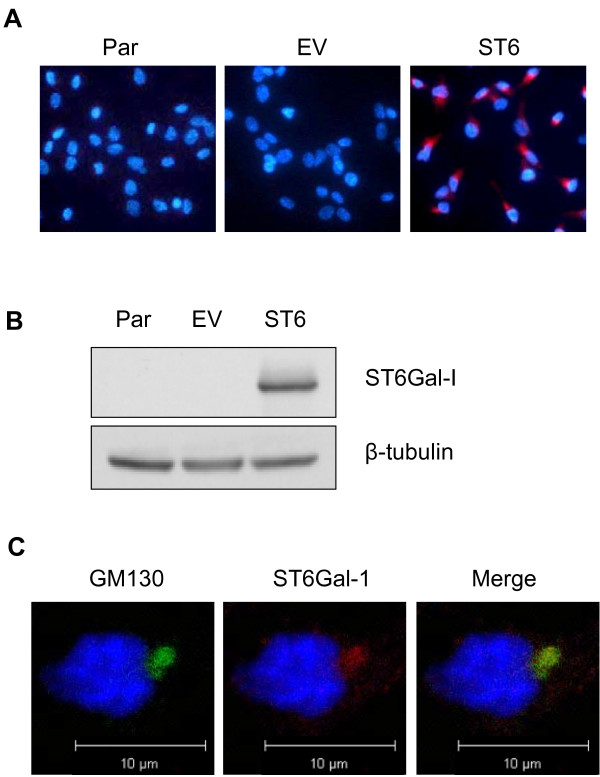
**ST6Gal-I expression in OV4 ovarian cancer cells.** OV4 cells that have no endogenous ST6Gal-I were stably transduced with either empty vector or ST6Gal-I-expressing lentivirus. ST6Gal-I expression was confirmed by immunocytochemistry (**A**) and immunoblotting (**B**). ST6Gal-I localization to the Golgi is shown by co-localization with the Golgi marker GM-130 in the ST6Gal-I forced expression line (**C**) Par = parental; EV = empty vector; ST6 = cells with forced ST6Gal-I expression.

In order to evaluate the effects of receptor α2-6 sialylation on tumor cell sensitivity to cisplatin, we utilized cell models with engineered ST6Gal-I expression. OV4 ovarian cancer cells have no detectable endogenous ST6Gal-I [13]; we therefore generated a stable cell line with forced ST6Gal-I expression. As shown in Figure 1A, the majority of cells with forced ST6Gal-I expression (ST6) exhibited high levels of immunostaining for ST6Gal-I, whereas none of the parental (Par) or empty vector-transduced (EV) cells were positive for ST6Gal-I. We confirmed forced ST6Gal-I expression by immunoblotting (Figure 1B). The pattern of staining was consistent with Golgi localization, evidenced by ST6Gal-I co-localization with the known Golgi protein, GM-130 (Figure 1C). The Golgi is the expected subcellular compartment for ST6Gal-I, demonstrating that ectopically expressed ST6Gal-I is correctly localized.

**Figure 2:**
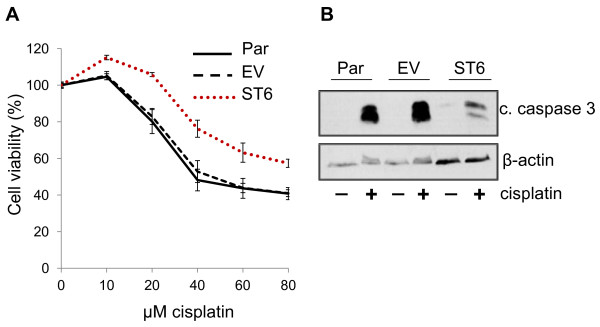
**Forced ST6Gal-I expression protects OV4 cells from cisplatin-induced cell death.** (**A**) Compared with Par or EV cells, OV4 cells with forced ST6Gal-I expression (ST6) exhibit attenuated cisplatin-induced cell death, as measured by ATP content. Means and S.E.M.s are shown for a representative experiment, with three independent experiments performed, and each experiment performed in triplicate. (**B**) ST6 cells have reduced levels of cleaved caspase-3 (representing caspase activation) following cisplatin treatment.

Par, EV and ST6 cells were treated with increasing doses of cisplatin and evaluated for cell viability by measuring ATP content. The ST6 cells maintained cell viability at higher cisplatin doses as compared with Par or EV cells (Figure 2A), indicating that ST6Gal-I-mediated sialylation protects against cell death. We also evaluated activation of caspase 3, the principal executioner caspase responsible for directing apoptosis. In concordance with cell viability assays, ST6 cells have decreased activation of caspase 3 as compared with Par or EV cells.

### Forced ST6Gal-I knockdown sensitizes tumor cells to cisplatin-induced cell death

**Figure 3:**
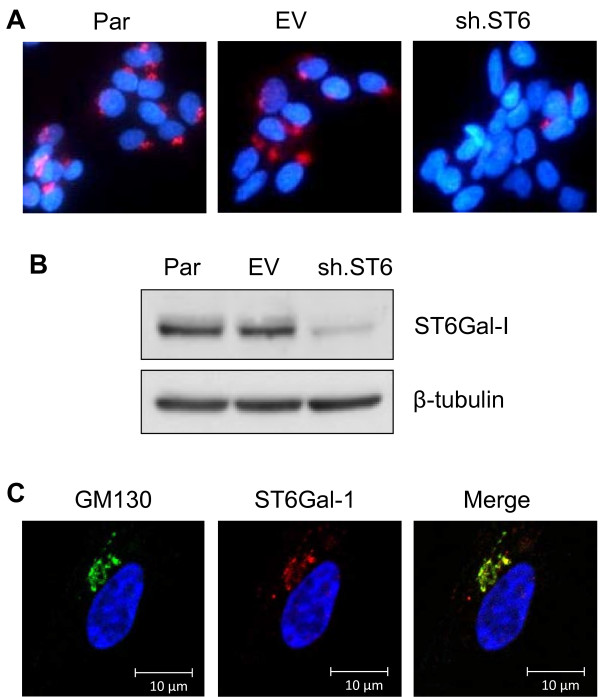
**ST6Gal-I knockdown in Pa-1 ovarian cancer cells.** Pa-1 cells that have high endogenous ST6Gal-I were stably transduced with either empty vector lentivirus, or virus expressing shRNA for ST6Gal-I. ST6Gal-I knockdown was confirmed by immunocytochemistry (**A**) and immunoblotting (**B**). ST6Gal-I localization to the Golgi was confirmed by co-localization with the Golgi marker GM-130 in the empty vector transduced line (**C**) Par = parental; EV = empty vector; sh.ST6 = cells stably expressing shRNA for ST6Gal-I.

**Figure 4:**
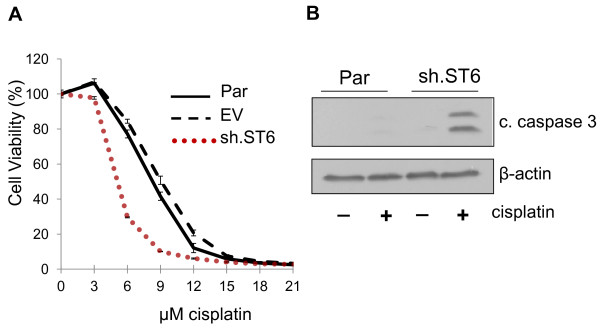
**shRNA-mediated ST6Gal-I knockdown sensitizes Pa-1 cells to cisplatin-induced cell death.** (**A**) Pa-1 cells with stable ST6Gal-I knockdown (sh.ST6) are sensitized to cisplatin-induced cell death, as measured by ATP content. Means and S.E.M.s are shown for a representative experiment, with three independent experiments performed, and each experiment performed in triplicate. (**B**) sh.ST6 cells have increased levels of cleaved caspase-3 following cisplatin treatment as compared with Par cells.

To further establish a role for ST6Gal-I in cisplatin sensitivity, ST6Gal-I expression was repressed by shRNA in Pa-1 ovarian cancer cells, a cell line with high endogenous levels of ST6Gal-I [13]. Effective knockdown of ST6Gal-I was confirmed by immunostaining and immunoblotting (Figure 3A and B). Golgi localization of endogenous ST6Gal-I is demonstrated by co-staining cells with GM-130 (Figure 3C). Parental (Par), empty vector (EV) and ST6Gal-I knockdown cells (sh.ST6) were exposed to increasing doses of cisplatin, and monitored for cell viability. As shown in Figure 4A, ST6Gal-I knockdown decreased the viability of Pa-1 cells, indicating enhanced sensitivity to cisplatin. Consistent with these results, sh.ST6 cells displayed greater activation of caspase 3 (Figure 4B).

While results shown in Figures 2 and 4 indicated that ST6Gal-I directly regulates cell response to cisplatin, it is interesting that higher concentrations were needed to achieve killing of OV4 cells, suggesting that OV4 cells have an inherent resistance to cisplatin that is independent of ST6Gal-I function. This observation is consistent with the extensive evidence indicating that tumor cells become resistant through many different mechanisms. Another factor to consider is that the efficacy of cell killing depends not only on the amount of cisplatin added extracellularly, but also on the rate of cisplatin uptake as well as intracellular metabolism of cisplatin. The intracellular half-life of cisplatin within OV4 and Pa-1 cells was not measured in the current study, therefore the relationship between intracellular and total cisplatin for the two cell lines is not known. Nonetheless, it is noteworthy that manipulating ST6Gal-I expression in an inherently resistant cell line (OV4) is still effective in regulating cisplatin response, supporting a causal role for ST6Gal-I in cisplatin sensitivity.

### Extended cisplatin treatment selects for ST6Gal-I expressing cells

**Figure 5:**
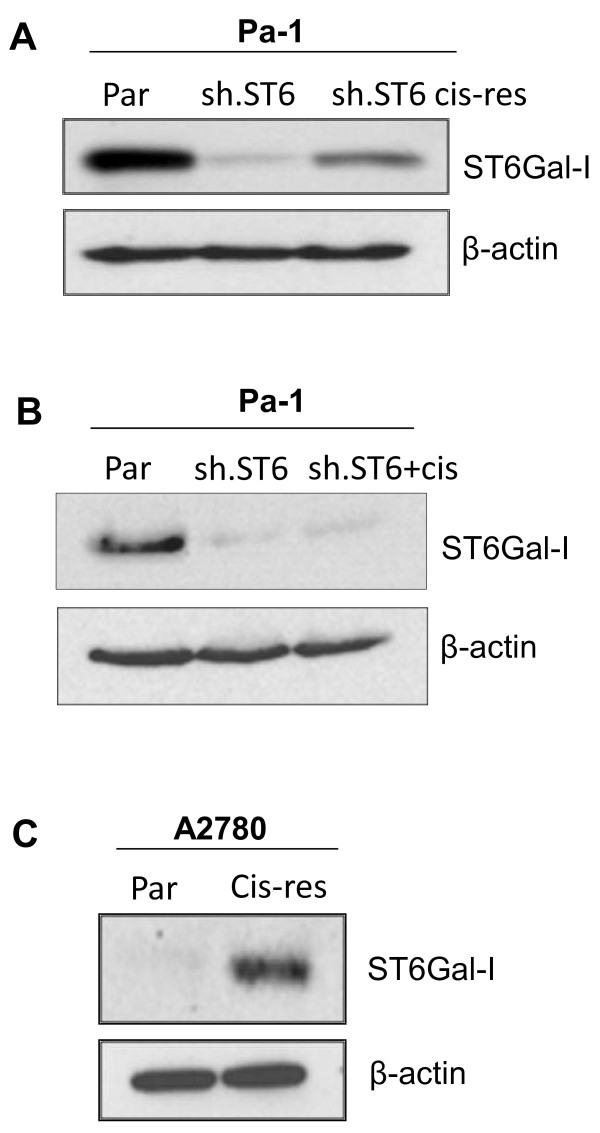
**Cells that are resistant to cisplatin have upregulated endogenous ST6Gal-I.** (**A**) Pa-1 cells with ST6Gal-I knockdown (sh.ST6) were exposed to cisplatin for 3 weeks, and the remaining viable population (sh.ST6 cis-res) was expanded and immunoblotted for ST6Gal-I. (**B**) Pa-1 cells with ST6Gal-I knockdown do not upregulate ST6Gal-I expression following a 24-hr treatment with cisplatin (sh.ST6 + cis). (**C**) Parental A2780 ovarian cancer cells (Par) and a cisplatin-resistant derivative population (Cis-res) were immunoblotted for endogenous ST6Gal-I.

Given that ST6Gal-I is upregulated in many types of cancer, including ovarian carcinoma [28], we hypothesized that cells with high ST6Gal-I expression may have a selective survival advantage. To address this hypothesis, we exposed Pa-1 cells with ST6Gal-I knockdown to prolonged low-dose cisplatin treatment. Notably, the Pa-1 sh.ST6 cell line represents a polyclonal cell population, and some variability in the degree of ST6Gal-I knockdown is observed among individual clones (as seen in Figure 3A). Pa-1 sh.ST6 cells were treated continuously with cisplatin for 3 weeks, during which greater than 90% of the cells were killed. The remaining viable population was then harvested, expanded, and immunoblotted for ST6Gal-I. As shown in Figure 5A, the viable cells exposed to cisplatin (sh.ST6 cis-res) had a higher level of ST6Gal-I, suggesting that ST6Gal-I conferred a survival benefit. To address the possibility that cisplatin may have induced ST6Gal-I expression, sh.ST6 cells were treated for 24 hours with the same dose of cisplatin (sh.ST6 + cis), however no changes in ST6Gal-I were observed (Figure 5B). These data suggest that the enhanced expression of ST6Gal-I in sh.ST6 cells treated with cisplatin for 3 weeks was due to selection for clones with higher ST6Gal-I, rather than induction of gene expression.

If the response of differentially-sialylated tumor cells to cisplatin treatment is indicative of a general protective effect of ST6Gal-I in the presence of cisplatin, then it would be expected that other cell models of cisplatin resistance may exhibit elevated ST6Gal-I expression. Thus, we immunoblotted for ST6Gal-I in the A2780 ovarian carcinoma cell line and its stably cisplatin-resistant derivative, A2780cp20 [27]. As shown in Figure 5C, a marked increase in ST6Gal-I expression was observed in the cisplatin-resistant A2780cp20 cells (Cis-res), providing further evidence of an association between ST6Gal-I upregulation and chemoresistance.

## Discussion

Despite the clear clinical significance of chemotherapy resistance, a single mechanism of resistance has not yet been established for all cases. The multifactorial nature of tumor cell resistance to cisplatin leaves open the possibility of novel mechanisms that remain undiscovered. In this study we show that forced expression of ST6Gal-I confers resistance to cisplatin, whereas ST6Gal-I knockdown conversely sensitizes cells to cisplatin. Furthermore, cells selected for resistance to cisplatin exhibit an upregulation in endogenous ST6Gal-I protein, suggesting that increased receptor α2-6 sialylation may provide tumor cells with a survival advantage. These findings illuminate a new mechanism for chemoresistance, and underscore the importance of the cellular glycosylation machinery in drug response. An aberrant glycan profile was one of the earliest identified characteristics of a cancer cell, and selective enrichment in α2-6 sialylation (relative to α2-3 sialylation), is a common feature of transformed cells [3]. It is also known that platinum drug-resistant cells have abnormal glycosylation [29,30], and studies spanning more than two decades indicate that cisplatin treatment alters the sialic acid content of tumor cells [31-33]. The link between these glycosylation changes and ST6Gal-I is currently unclear, however the inhibitory effect of ST6Gal-I on cisplatin-induced cell death is likely driven by the activity of variantly-sialylated surface receptors, given that ST6Gal-I modifies glycoproteins bound for the plasma membrane or secretion (and not cytosolic proteins). Interestingly, tumors expressing activating ras mutations [34] or ras overexpression [35] are typically resistant to cisplatin, and ST6Gal-I is one of the targets upregulated by ras signaling [9,10]. Furthermore, we recently reported that high ST6Gal-I expression correlates with expression of the cancer stem cell markers ALDH1 and CD133, suggesting that ST6Gal-I activity may contribute to stem-like cell behaviors including chemoresistance [36].

One predominant surface receptor known to modulate cisplatin sensitivity is the Fas death receptor. Fas is activated by binding to FasL, which in turn causes receptor internalization, formation of the Death Inducing Signaling Complex (DISC), followed by activation of apoptotic caspases. Caspase activation is also a critical downstream event following cisplatin-induced DNA damage, and cisplatin-resistant cells exhibit attenuated activation of caspases 3, 8 and 9 [35]. Cisplatin is reported to cause clustering and activation of the Fas receptor in a ligand independent manner [36], as well as increased Fas expression [37-43]. Additionally, cisplatin stimulates the aggregation of Fas into lipid rafts [44,45], which is correspondingly important for Fas internalization and apoptotic signaling [46]. In mice with subcutaneous tumors formed from syngeneic Lewis lung carcinoma cells, one intraperitoneal dose of cisplatin induced a dramatic increase in Fas expression in the tumors, and also stimulated tumor regression [43]. In this same study, the anti-tumor effects of cisplatin were abrogated in mice deficient in FasL. These results implicate cisplatin-induced Fas upregulation in promoting tumor cell death [43], and further suggest that in order to acquire cisplatin-resistance, tumor cells may evolve mechanisms to disable Fas signaling. Our prior studies demonstrated that Fas is a ST6Gal-I substrate, and that increased α2-6 sialylation of Fas functions to inhibit Fas receptor internalization and DISC formation [8], effectively shutting off Fas apoptotic signaling. Hence, α2-6 sialylated Fas isoforms could play a part in cisplatin resistance.

Another potential mechanism for ST6Gal-I-mediated cisplatin-resistance may involve the differential sialylation of one or more drug transporters. Many cisplatin-resistant cell lines show reduced accumulation of cisplatin [47], pointing to dysfunctions in cell surface transporters that control either drug uptake or efflux. Defective glycosylation of ATP binding cassette (ABC) transporters has been suggested to contribute to cancer development, and possibly, chemoresistance [48]. Liang et al. reported that in epidermoid carcinoma cells selected for resistance to cisplatin, the MRP1 transporter (also known as ABCC1) was aberrantly glycosylated, and this was associated with mislocalization to intracellular compartments and reduced cell surface expression [29]. Similarly, altered N-glycosylation of MRP1 and MRP4 was correlated with cisplatin and oxaliplatin resistance in ovarian cancer cells [49]. In this latter study, *N*-glycosylation defects were linked to reduced levels of two glycosyltransferases: (i) N-acetylglucosamine-1-phosphate transferase, gamma subunit (GNPTG) and (ii) mannosyl (alpha-1,6)-glycoprotein beta-1,6-N-acetyl-glucosaminyltransferase (MGAT5) [49]. *N*-glycans are also known to be crucial for the stability of the ABCG2 transporter in the endoplasmic reticulum [30,50]. These findings indicate the importance of glycosylation in transporter function, and suggest that studies of variant transporter sialylation may be a fruitful area for future research.

Although the mechanisms underlying the effects of ST6Gal-I activity on cisplatin sensitivity are not yet understood, the current study adds to the body of literature implicating this enzyme as a major contributor to tumor cell survival. In addition to conferring cisplatin resistance, ST6Gal-I-mediated receptor sialylation blocks apoptotic signaling by the Fas [8] and TNFR1 [19] death receptors, and also inhibits galectin-induced cell death [16-18]. Taken together, these results suggest that ST6Gal-I may be a promising clinical target, and that inhibition of ST6Gal-I expression or activity could be employed to sensitize tumor cells to platinum drugs, increasing therapeutic efficacy.

## Conclusions

We demonstrate that ST6Gal-I expression in ovarian tumor cells confers resistance to cisplatin-mediated cell death and that cell lines selected for resistance to cisplatin strongly express ST6Gal-I. Hence, tumor cell expression of ST6Gal-I possibly contributes to chemotherapy resistance in a clinical setting. This finding points to the potential of targeting ST6Gal-I in ovarian cancer treatment and identifies ST6Gal-I as a novel contributor to cisplatin resistance.

## Competing interests

The authors have no conflicts to disclose.

## Authors’ contributions

MJS developed the methodology, acquired and interpreted data, and drafted the manuscript. AFS and JWW acquired data. ESS acquired data and aided in study design. CNL conceived and designed the study. SLB conceived and designed the study, developed the methodology, interpreted data, edited the manuscript, and oversaw the study. All authors have read and approved the final manuscript.
